# ﻿Resurrection of *Pleurospermumlecomteanum* H.Wolff (Apiaceae) based on molecular and morphological evidence

**DOI:** 10.3897/phytokeys.212.94280

**Published:** 2022-10-24

**Authors:** Jing Zhou, Junmei Niu, Xinyue Wang, Shilin Zhou, Zhenwen Liu

**Affiliations:** 1 School of Pharmaceutical Science and Yunnan Key Laboratory of Pharmacology for Natural Products, Kunming Medical University, Kunming 650500, China Kunming Medical University Kunming China; 2 Yunnan Academy of Forestry and Grassland, Kunming 650201, China Yunnan Academy of Forestry and Grassland Kunming China; 3 Gaoligong Mountain, Forest Ecosystem, Observation and Research Station of Yunnan Province, Yunnan, China Gaoligong Mountain, Forest Ecosystem, Observation and Research Station of Yunnan Province Yunnan China; 4 Yunnan Key Laboratory of Biodiversity and Ecological Security of Gaoligong Mountain, Yunnan, China Yunnan Key Laboratory of Biodiversity and Ecological Security of Gaoligong Mountain Yunnan China

**Keywords:** Apiaceae, *Pleurospermum*, resurrection, synonym, taxonomy

## Abstract

The taxonomic placement of *Pleurospermumlecomteanum*, previously synonymized with *Pleurospermumwilsonii*, was carefully examined using herbarium specimens and molecular evidence. The results showed that *Pleurospermumlecomteanum* is distinguished from *P.wilsonii* by several morphological characters. Its phylogenetic position is separate from *P.wilsonii* in the ML tree. Therefore, *Pleurospermumlecomteanum* should be restored as a distinct species.

## ﻿Introduction

Of the four major worldwide distribution centers of Apiaceae, China has the highest taxonomic diversity at the species level (614–657 species), and represents approximately 1/5 of all species recognized within the family (Sheh and Shu 1987; Sheh et al. 2005; Pimenov 2017). However, numerous species in Chinese Apiaceae remain rather enigmatic and have not been investigated adequately, even morphologically, because of their remote distribution and inadequate number of collections (Zhou et al. 2008; Pimenov 2017). Therefore, in the past few years, large efforts have been devoted to field investigations and examination of herbarium specimens towards a comprehensive understanding of the species of Chinese Apiaceae.

Until recently, *Pleurospermum* Hoffm. was treated as comprising about 50 species widely distributed in northern Asia and East Europe, of which 39 were in China (Pan and Watson 2005). Taxonomic changes by Pimenov and Kljuykov (2000a, b), Pimenov (2017), Pimenov et al. (2000) and Zhou et al. (2020a, 2021), have reduced that number considerably. However, relationships among some synonymous species are still ambiguous.

This statement also belongs to the widely accepted *Pleurospermumwilsonii* H.de Boissieu. It was described by H.de Boissieu based on the collections from western China in 1906. In the past years, several taxa have been included within it, namely *Physospermopsislalabhduriana* Farille & S.B.Malla, *Pleurospermumcnidiifolium* H.Wolff, *P.crassicaule* H.Wolff, *P.lecomteanum* H.Wolff, *P.tanacetifolium* H.Wolff and *P.thalictrifolium* H.Wolff (Shan and Sheh 1979; Pan and Watson 2005; Pimenov 2017). *Pleurospermumtanacetifolium* and *P.thalictrifolium* were later merged with *Pleurospermumdavidii* Franch. and *Pleurospermumastrantioideum* (H.de Boissieu) K.T.Fu et Y.C.Ho, respectively (Pimenov 2017). Pimenov and Kljuykov (2000a) have since transferred *Pleurospermumwilsonii*, *P.davidii* and *P.astrantioideum* to *Hymenidium*, but these taxonomic novelties need further confirmation based on the extensive molecular analysis (Zhou et al. unpublished data).

During our fieldwork in western China, we discovered several populations with morphological characters that are different from *Pleurospermumwilsonii* (10–25 cm tall, stem sometimes shortened vs. 15–60 cm tall, stem elongated; 2–3-pinnatisect, ultimate segments narrowly ovate or lanceolate, entire or 2–3-lobed vs. 1-pinnate, or subbipinnatisect with ultimate segments ovate or suborbicular, base cuneate, margins irregularly serrate to deeply lobed; rays 8–15, unequal or equal vs. 10–25, subequal). After consulting the relevant protologues (Boissieu 1906; Wolff 1925, 1926, 1929; Farille et al. 1985) and type specimens for each of the names, we consider that the population from Chayu County, Tibet, is identical with *P.wilsonii*, while the populations from Sichuan and Qinghai provinces correspond to *P.lecomteanum*, based on the morphology. A further analysis of comparative DNA sequences is presented here to clarify the taxonomic relationships between *P.lecomteanum* and *P.wilsonii*, and to identify their potential close relatives within the molecular framework of Apiaceae subfamily Apioideae.

## ﻿Materials and methods ﻿

### ﻿Morphological analysis

**Figure 1.**
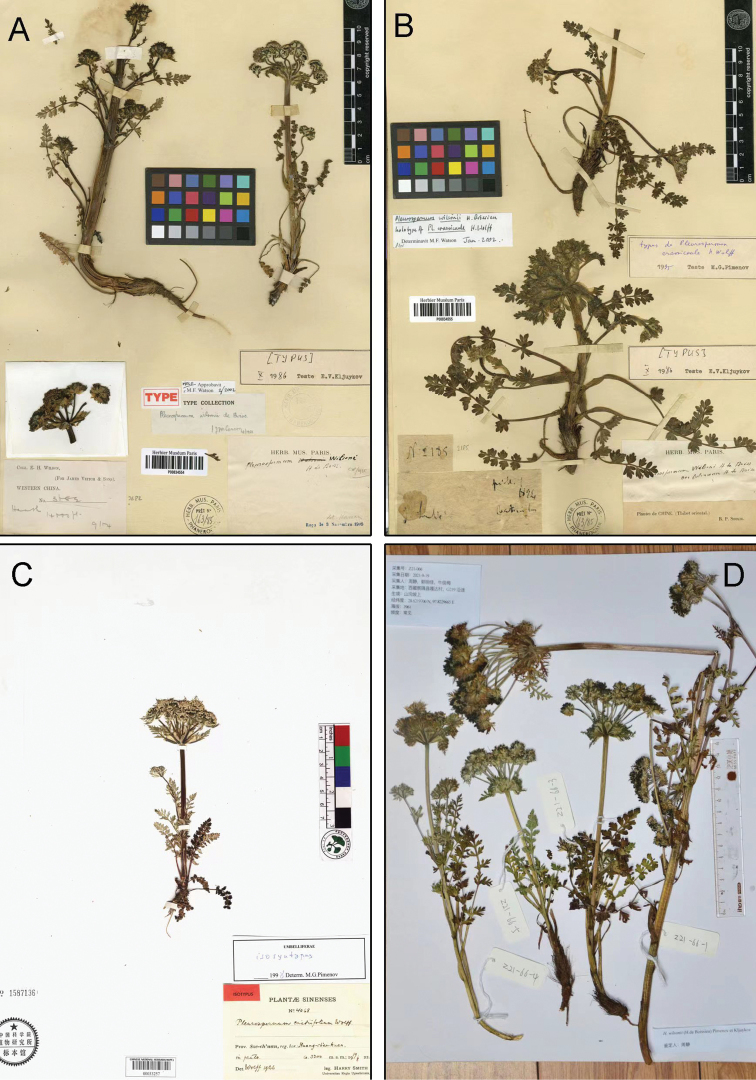
**A** lectotype of *Pleurospermumwilsonii* from P (P00834554) **B** holotype of *Pleurospermumcrassicaule* from P (P00834555) **C** syntype of *Pleurospermumcnidiifolium* from PE (PE00033257) **D** the voucher specimen of *Pleurospermumwilsonii* from Z21-066.

**Figure 2.**
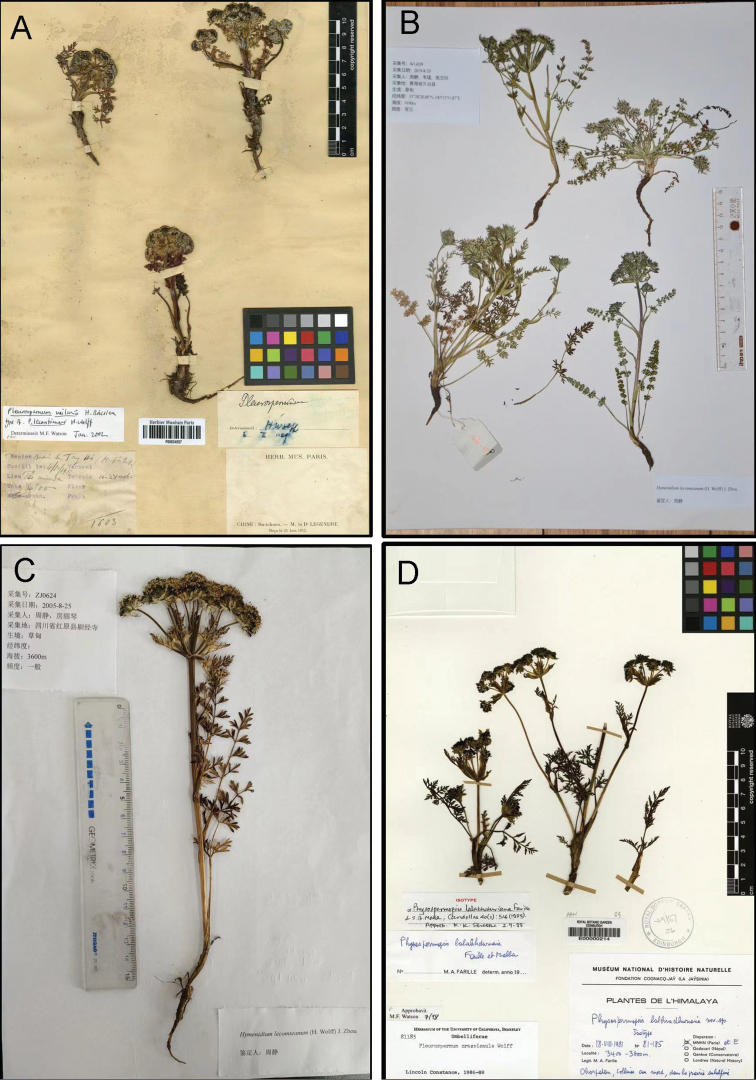
**A** syntype of *Pleurospermumlecomteanum* from P (P00834557) **B** the voucher specimen of *P.lecomteanum* from WG039 **C** the voucher specimen of *P.lecomteanum* from ZJ0624 **D** isotype of *Physospermopsislalabhduriana* from E (E00000214).

Digital resources of CVH, GBIF and JSTOR Global Plants for the type specimens of *Pleurospermumwilsonii* (K000685336, P00834554) and its synonyms (*Physospermopsislalabhduriana*, E00000214; *Pleurospermumcnidiifolium*, PE00033257; *Pleurospermumcrassicaule*, P00834555; *Pleurospermumlecomteanum*, P00834556, P00834557) were carefully examined (Figs 1, 2). The morphological characters of *P.lecomteanum* were examined based on the types and specimens we collected in the field. The fruit was studied using a stereo microscope. Morphological comparisons between *P.wilsonii* and *P.lecomteanum* are provided in Table 1.

**Table 1.** Morphological comparison between *Pleurospermumwilsonii* and *P.lecomteanum*.

Character	*P.lecomteanum*	*P.wilsonii*
Stem	10–25 cm tall, sometimes shortened	15–60 cm tall, elongated
Leaf	Oblong in outline, 2–3-pinnatisect	Oblong-lanceolate in outline, 1-pinnate or subbipinnatisect
Pinnae	4–8 pairs, shortly petiolulate or subsessile	5–9 pairs, sessile
Ultimate segment	Narrowly ovate or lanceolate, 3–5× 1–1.5 mm, entire or 2–3-lobed	Ovate or suborbicular, 7–14×4–10 mm, base cuneate, margins irregularly serrate to deeply lobed
Ray	8–15, unequal or equal	10–25, subequal
Calyx	Triangular	Triangular-ovate
Vitta	1 in each furrow, 2 on commissure	1–2 in each furrow, 2 on commissure

### ﻿Phylogenetic analysis

The new nrDNA ITS sequences for five accessions of *P.wilsonii* and four accessions of *P.lecomteanum* (Table 2) were generated with the protocols described by Zhou et al. (2008). The new sequences were then aligned with the matrix of Zhou et al. (2020b) using the BioEdit sequence alignment editor (Hall 1999). All sequences were used to infer phylogenetic relationships. A maximum likelihood (ML) analysis was conducted with RAxML v.8.2.4 (Stamatakis 2006), using the GTR +G substitution model with 1000 bootstrap replicates, with other parameters following the default settings.

**Table 2.** Voucher information and GenBank accession numbers for the five accessions of *Pleurospermumwilsonii* and four accessions of *P.lecomteanum* used in the phylogenetic analysis.

Taxa	Source/Voucher	GenBank number
*Pleurospermumwilsonii* H.de Boissieu	China, Xizang, Chayu, Z21-66-3 (KUN)	ON715443
	China, Xizang, Chayu, Z21-66-5 (KUN)	ON715444
	China, Xizang, Chayu, Z21-66-4 (KUN)	ON715445
	China, Xizang, Chayu, Z21-66-1 (KUN)	ON715446
	China, Xizang, Chayu, Z21-66-2 (KUN)	ON715447
*P.lecomteanum* H.Wolff	China, Qinghai, Jiuzhi, WG034 (KUN)	ON715451
	China, Qinghai, Jiuzhi, WG036 (KUN)	ON715450
	China, Qinghai, Jiuzhi, WG037 (KUN)	ON715449
	China, Qinghai, Jiuzhi, WG039 (KUN)	ON715448

## ﻿Results and discussion

**Figure 3.**
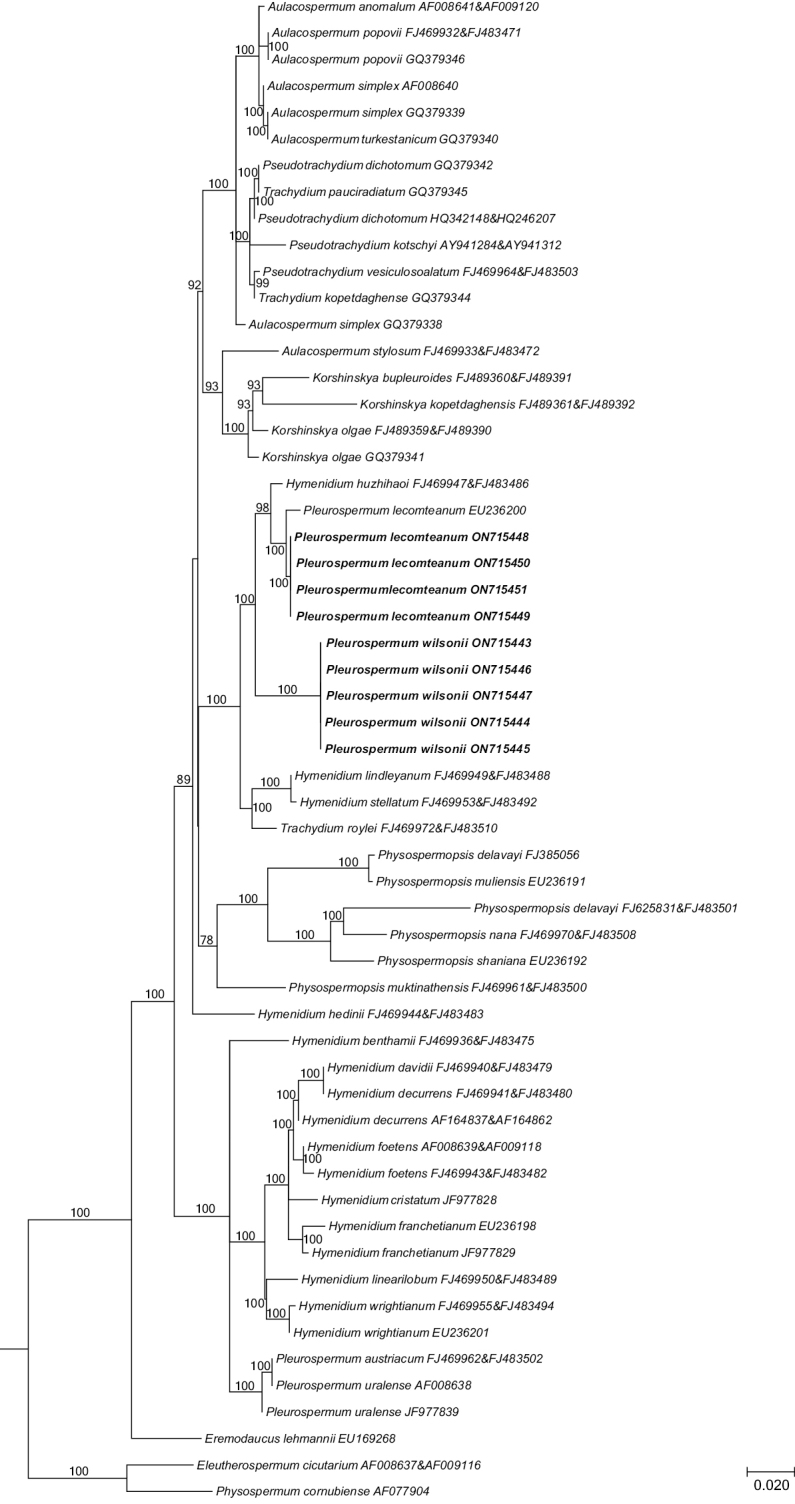
The consensus tree obtained from maximum likelihood analysis of 2920 nrDNA ITS sequences from Apiaceae subfamily Apioideae showing the tribe Pleurospermeae, with support values (≥50%) provided next to the branches. The nine newly sampled accessions are shown in bold.

The phylogenetic results revealed that all accessions of *Pleurospermumwilsonii* allied together, and constituted a sister group relationship with the clade of *Hymenidiumhuzhihaoi* Pimenov & Kljuykov and *P.lecomteanum* (Fig. 3, the complete tree containing all sampled representatives is available upon request). The whole clade fell within the tribe Pleurospermeae, and showed close relationship with the clade of *Hymenidiumlindleyanum* (Klotzsch) Pimenov & Kljuykov, *Hymenidiumstellatum* (D.Don) Pimenov & Kljuykov and *Trachydiumroylei* Lindley. The pairwise sequence divergence value between *P.wilsonii* and *P.lecomteanum* was 3.44%.

Recently, the circumscription of *Pleurospermum* was reduced to comprise only two species (the type species *P.austriacum* L., and *P.uralense* Hoffm.), while the other species were referred to *Aulacospermum*, *Hymenidium*, *Hymenolaena*, *Physospermopsis*, and *Pterocyclus* (Pimenov and Kljuykov 2000a, 2000b). However, only two of these genera, *Aulacospermum* and *Hymenolaena*, were supported as monophyletic groups in the molecular phylogenetic study by Valiejo-Roman et al. (2012). *Hymenidium* seems to be non-monophyletic, with its members assigned to the *Acronema* clade, the East-Asia clade, the *Sinodielsia* clade, the *Pleurospermopsis* clade and the Pleurospermeae (Zhou et al. 2009, 2020b; Wei 2020). Furthermore, the species of *Hymenidium* did not ally as monophyletic within three of the above clades. Pimenov & Kljuykov (2000a) indicated that *Hymenidium* in current circumscription is a genus with ambiguous taxonomy, and probably comprised several more distinct species groups. Recently, we conducted molecular phylogenetic studies for *Pleurospermum* and related genera, in which we sorted members of *Pleurospermum* into major clades of Apioideae, assessed relationships of these members to other apioid taxa within each of these major clades (Wei 2020), transferred *Pleurospermumbicolor* (Franch.) C.Norman ex Z.H.Pan & M.F.Watson into the genus *Pleurospermopsis* as *Pleurospermopsisbicolor* (Franch.) J.Zhou & J.Wei (Zhou et al. 2020a), confirmed the status of *Pterocyclus* as a separate genus with four species (*Pterocyclusangelicoides* (Wallich ex DC.) Klotzsch, *P.rotundatus* (DC.) Pimenov & Kljuykov, *P.forrestii* (Diels) Pimenov & Kljuykov, and a restored species, *P.wolffianus* Fedde ex H.Wolff; Zhou et al. 2021). All of these studies have enhanced our understanding of *Pleurospermum* and related genera, and brought us one step closer towards a more natural classification system.

*Pleurospermumlecomteanum* was described by H.Wolff based on collections from China in 1929. In Flora Reipublicae Popularis Sinicae, along with *P.cnidiifolium*, *P.tanacetifolium* and *P.thalictrifolium*, it was synonymized with *P.crassicaule* (Shan and Sheh 1979). All of the above species, plus *Physospermopsislalabhduriana*, were included within *P.wilsonii* in the Flora of China (Pan and Watson 2005). After consulting the types of *P.tanacetifolium* (GB0048823) and *P.thalictrifolium* (GB0048825), we consider that it is reasonable to merge them with *P.davidii* and *P.astrantioideum*, respectively as proposed by Pimenov (2017). *Physospermopsislalabhduriana* was described based on specimens from Nepal in 1985 (Farille et al. 1985). The morphology of the leaf and bracteoles of the isotype (E00000214) is different from that of *P.wilsonii*. It should be regarded as a distinct species that is not distributed in China (Pimenov 2017). Among the names treated as synonyms of *P.wilsonii*, *Pleurospermumcrassicaule* and *P.cnidiifolium* were each described by H.Wolff in 1925 and 1926, respectively. We have examined their type materials (holotype P00834555 for *P.crassicaule* and syntype PE00033257 for *P.cnidiifolium*), and consider that they cannot be separated from *P.wilsonii* and should be merged into a single species. However, we found a set of morphological characteristics, including the stem length, shape and division of leaves and pinnae, as well as the number and length of rays (Table 1), distinguished *P.lecomteanum* from *P.wilsonii*. In our field investigation in Qinghai and Sichuan provinces, we collected several specimens of *P.lecomteanum*, whose morphology is exactly the same as the type (P00834556 and P00834557), that led us to observe its morphology more carefully and reassess the status of this taxon. In our molecular analysis, the accessions of *P.lecomteanum* allied as monophyletic, and comprised a sister group relationship with *Hymenidiumhuzhihaoi*.

*Hymenidiumhuzhihaoi* was a species recently described by Pimenov and Kljuykov (Pimenov and Kljuykov 2004). It was distinguished from *P.lecomteanum* by being subacaulescent, umbellules compact, and apex of bracteoles 3–10-lobed, or rarely entire, and by its 1.23% nucleotide divergence. Therefore, both morphological and molecular evidence support recognition of *P.lecomteanum* as a distinct species. Since *Hymendium* is a polyphyletic genus need to be further revision, and its type species (*H.brunonis* (DC.) Lindl.) does not fall into the Pleurospermeae to which *P.lecomteanum* belongs, so we here merely restore it as a distinct species without further taxonomic treatment.

### ﻿Taxonomy

#### ﻿ Pleurospermum lecomteanum

Taxon classificationPlantaeApialesApiaceae

H.Wolff, 1929, Repert. Spec. Nov. Regni Veg. 27: 116.

D913EF5A-0A55-5A08-8F49-674FE8016A7B

##### Type.

China. Su-tchuen [Sichuan]: Bassin de Tongho (M.Thibet), Dzenla, roches metamorph., 3500 m, prairies alpines, 24 September 1911, *A.F. Legendre 1537* (lectotype P! barcode P00834556, designated by Pimenov, Kljuykov, 2000a: 550); Su-tchuen [Sichuan]: Bassin de Tong-ho (M.Thibet) Tse minuda, terrain schistos., 4500 m, 04 October 1911, *A.F. Legendre 1603* (syntype P! barcode P00834557); Sze-ch’uan [Sichuan]: reg. bor.-occid., Dalgang cia. 50 km VSV von Merge, 3500 m, 03 September 1922, *H. Smith 4313* (lectotype GB! barcode GB0048821; isolectotype UPS!).

##### Other specimens examined.

China. Qinghai: Jiuzhi, 4100 m, 20 August 2019, *J. Zhou*, *J. Wei & Y.Z. Gao G034*, *G036*, *G037*, *G039* (KUN); Sichuan: Hongyuan, 3600 m, 25 August 2005, *J. Zhou & L.Q. Fang ZJ0624* (KUN).

##### Description.

Herbs perennial, 10–25 cm tall. Taproot long conic, simple. Stem erect, ribbed, sometimes shortened, bases with remnant sheaths. Basal and lower leaves petiolate; petioles 3–6 cm, petiole base sheathing, oblong, ca. 1.5–2 cm long; blade oblong in outline, 2–3-pinnatisect; pinnae 4–8 pairs, short petiolulate or subsessile; ultimate segment narrowly ovate or lanceolate, 3–5× 1–1.5 mm, entire or 2–3-lobed. Upper leaves smaller and reduced, sheath prominent. Umbels compound, terminal or lateral; bracts 4–6, leaf-like, 2–6 cm long; rays 8–15, 1–7 cm long, unequal or equal; bracteoles 6–8, broadly ovate, similar to bracts, margin broadly white membranous, apex pinnate, longer than the flowers. Calyx teeth triangular, 0.5 mm long. Petals white, or purple, oblong-obovate, apex acute, short incurved. Stylopodium flat; styles longer than stylopodium. Fruit oblong, slightly compressed laterally; ribs prominent, broadly winged; vittae 1 in each furrow, 2 on commissure. Fl. Aug–Sep, fr. Sep–Oct.

##### Note.

In flora of China, *Pleurospermumwilsonii* was described as 2–3-ternate-pinnate (Pan and Watson 2005). However, the type specimens and the protologues for it and its synonymous species, *P.crassicaule*, show that their leaf blades are “simpliciter pinnata” or “1- vel subbipinnatisecta” (Boissieu 1906; Wolff 1925). For *Pleurospermumcnidiifolium*, H. Wolff described its blades as “bi- vel subtripinnatipartita”, but the type has 1-pinnate (PE00033257), or 2-pinnatisect (GB0048821). After careful examination of GB0048821, we considered that its morphology was more similar to *P.lecomteanum.* That is, for *P.cnidiifolium*, the type PE00033257 should be selected, while GB0048821 should be put under *P.lecomteanum*. Our population in Chayu County of Tibet with variable leaf morphology (1-pinnate or subbipinnatisect) yielded identical ITS sequences. Therefore, with the resurrection of *P.lecomteanum*, the description for *P.wilsonii* should be revised as: blades 1-pinnate or subbipinnatisect, ultimate segments ovate or suborbicular, 7–14×4–10 mm, base cuneate, margins irregularly serrate to deeply lobed, rays 10–25, subequal. Furthermore, *Pleurospermumlecomteanum* occurs in the open grasslands in Yunnan, Sichuan, Gansu and Qinghai provinces of China, while *P.wilsonii* is on the south slope of mountains in Sichuan, Qinghai and Xizang provinces of China.

### ﻿Key to *Pleurospermumwilsonii* and *P.lecomteanum*

**Table:** 

1	Leaf blades 1-pinnate or subbipinnatisect, ultimate segments ovate or suborbicular, 7–14×4–10 mm, base cuneate, margins irregularly serrate to deeply lobed	***P.wilsonii***
–	Leaf blades 2–3-pinnatisect, ultimate segments narrowly ovate or lanceolate, 3–5× 1–1.5 mm, entire or 2–3-lobed	***P.lecomteanum***

## Supplementary Material

XML Treatment for Pleurospermum lecomteanum
